# Development of a Risk Score for AKI onset in COVID-19 Patients: COV-AKI Score

**DOI:** 10.1186/s12882-023-03095-4

**Published:** 2023-03-02

**Authors:** Henrique Palomba, Daniel Cubos, Fernando Bozza, Fernando Godinho Zampieri, Thiago Gomes Romano

**Affiliations:** 1 Hospital Vila Nova Star – ICU and Critical Care Nephrology Department, Rua Dr. Alceu de Campos Rodrigues 126, São Paulo, Brazil; 2 Instituto D’Or de Pesquisa e Ensino, Avenida República do Líbano 611, São Paulo, Brazil; 3 Instituto Nacional de Infectologia Evandro Chagas Fundação Oswaldo Cruz FIOCRUZ, Avenida Brasil 4365 , Rio de Janeiro, Brazil; 4 Hospital São Luiz Itaim - Oncologic Critical Care Department, Rua Dr. Alceu de Campos Rodrigues 95, São Paulo, Brazil; 5 ABC Medical School Nephrology Department Assistant Professor, Avenida Príncipe de Gales 821, Santo André, Brazil

## Abstract

**Purpose:**

Acute Kidney Injury (AKI) in COVID-19 patients is associated with increased morbidity and mortality. In the present study, we aimed to develop a prognostic score to predict AKI development in these patients.

**Materials and methods:**

This was a retrospective observational study of 2334 COVID 19 patients admitted to 23 different hospitals in Brazil, between January 10th and August 30rd, 2020. The primary outcome of AKI was defined as any increase in serum creatinine (SCr) by 0.3 mg/dL within 48 h or a change in SCr by ≥ 1.5 times of baseline within 1 week, based on Kidney Disease Improving Global Outcomes (KDIGO) guidelines. All patients aged ≥ 18 y/o admitted with confirmed SARS-COV-2 infection were included. Discrimination of variables was calculated by the Receiver Operator Characteristic Curve (ROC curve) utilizing area under curve. Some continuous variables were categorized through ROC curve. The cutoff points were calculated using the value with the best sensitivity and specificity.

**Results:**

A total of 1131 patients with COVID-19 admitted to the ICU were included. Patients mean age was 52 ± 15,8 y/o., with a prevalence of males 60% (*n* = 678). The risk of AKI was 33% (*n* = 376), 78% (*n* = 293) of which did not require dialysis. Overall mortality was 11% (*n* = 127), while for AKI patients, mortality rate was 21% (*n* = 80). Variables selected for the logistic regression model and inclusion in the final prognostic score were the following: age, diabetes, ACEis, ARBs, chronic kidney disease and hypertension.

**Conclusion:**

AKI development in COVID 19 patients is accurately predicted by common clinical variables, allowing early interventions to attenuate the impact of AKI in these patients.

## Introduction

SARS-COV-2 was initially described in December 2019 in Wuhan, China and rapidly escalated to a pandemic in March 2020 [1, 2]. Since this time, our understanding of COVID-19 disease has evolved, but the natural history of this serious disease has not changed. The increasingly recognized systemic involvements such as acute kidney injury (AKI), stroke and myocardial injury contributes to the complexity and poor outcomes in many patients [3–6].

AKI is of particular interest and is associated with greater incidence, morbidity and mortality in COVID-19 patients, especially in cases with serious pulmonary disease and need for mechanical ventilation, probably as a result of the kidney-lung relationship previously described by other authors [7, 8]. Recently, some authors observed an incidence of AKI in COVID-19 patients varying from 25 to 57%, depending on the population studied and AKI criteria, with higher incidences described in critically ill patients [9, 10]. Numerous studies attempted to characterize the clinical course of renal disease in COVID-19 patients, but the results are variable [10–14].

Based on numerous studies, researchers believe that angiotensin-converting enzyme (ACE2) receptor serves as a co-transporter for SARS-COV-2 to enter the cells. Although the etiology of renal damage in patients with COVID-19 is multifatorial and associated with multiple mechanisms such as hypovolemia, rhabdomyolysis, microthrombosis, inflammation and virus-induced damage to tubular cells [15–17], it is believed that ACE2 receptors may play a role, given the high affinity of SARS-COV-2 for ACE2 receptors presented at high concentrations in the brush borders of renal tubular epitelial cells and medications such as angiotensin-converting enzyme (ACE) or angiotensin receptor blocker (ARB) may predispose or protect against AKI development in these patients [18–24].

Recently some prognostic scores to predict the development of critical illness and need for mechanical ventilation have been proposed and validated for COVID-19 patients. Unfortunately, the natural course of AKI development were not mentioned in these studies, precluding any clinical decision about the profile of AKI patients during hospitalization for COVID-19 [25, 26].

The objective of this study was to describe the risk factors associated with AKI in COVID 19 patients admitted to Intensive Care Unit (ICU) and to develop a specific prognostic score with clinical variables (including ACE and ARB use) that could accurately identify high-risk patients for AKI development during hospitalization for COVID-19 related complications.

## Materials and methods

This was a retrospective observational study of 2334 COVID-19 patients admitted to 23 different private hospitals in Brazil, between January 10th and August 30rd, 2020. The study was approved by the National Teaching and Ethical Committee (CONEP) under number: 29496920.8.0000.5262 and informed consent was waived due to the observational nature of the study.

The primary outcome of AKI was defined as any increase in serum creatinine (SCr) by 0.3 mg/dL within 48 h or a change in SCr by ≥ 1.5 times of baseline within 1 week, based on Kidney Disease Improving Global Outcomes (KDIGO) guidelines. The lowest SCr reading during hospitalization was used as the baseline for AKI definition. The staging of AKI was also defined according to the KDIGO criteria [27]. We did not use the urine output criteria to define AKI as the documentation of urine output in the electronic health record was unreliable. Chronic Kidney Disease (CKD) was defined as the glomerular filtration rate < 60 mL/min using the CKD-Epidemiology Collaboration equation. Patients on chronic renal replacement therapy were excluded from this analysis.

All patients aged ≥ 18 y/o admitted with confirmed SARS-COV-2 infection were consecutively included. We defined confirmed infection as positive reverse transcriptase-polymerase chain reaction (RT-PCR) from a nasal or throat swab together with clinical symptons or radiological findings suggestive of COVID-19 infection. Data were collected by a highly trained team and included demographic data such as comorbidities and use of medications, in addition to laboratory data at the time of ICU admission and outcomes such as need for mechanical ventilation or vasopressors, hospital discharge and death.

### Statistical analysis

The continuous variables were submitted to Kolmogorov–Smirnov and Shapiro–Wilk tests and their values were expressed as median and 25th/75th percentiles or as mean and standard deviation for parametric and non-parametric variables, respectively.

The categorical variables were submitted to Pearson’s chi square or Fisher’s exact test, if applicable, and were presented as absolute values and percentages. Discrimination of was calculated using the ROC (Receiver Operating Characteristic) curve and the categorization of some continuous variables was also performed using the ROC curve. The cut-off scores for each clinical variable were calculated using the p value with the best specificity and sensitivity for that particular variable.

The candidate variables for the multivariable model were included at the p-value of 0.2 in the univariable analysis and a step-by-step method was used to select each significant variable for the final logistic regression model, with the calculations of corresponding adjusted odds ratios (ORs) and 95% confidence intervals (CI).

The AKI Score variables were those that resulted from the COX Regression, being excluded one by one (step by step) until all predictor variables reached significance. The candidate variables for the model were those that reached significance as a value of p <  = 0.2 on the Kaplan–Meier Survival Curve using the Log-Rank test. The cutoff points of the continuous variables used in the AKI score were obtained through the ROC curve, corresponding to the highest value of the sum of sensitivity + specificity of all possible cut-off points obtained on the curve.

Variables with missing data up to 10% were: ACEi, ARB, Hypertension and Platelets. Variables with missing data from 10.1% to 20% were: Sodium, Potassium, pH and PCO2. There were no variables with more than 20% missing data of clinical relevance. After this classification, the following criterion was adopted: Variables with up to 10% of missing data were not considered influential and variables with missing data between 10.1% and 20% were performed Canonical Correlation (Canonical Analyses) with Wilk's lambda test to investigate possible biases of association of the missing data with the other variables and with the outcome of the study. Finally, all missing data were submitted to the “Full informationmaximum-likelihood (FIML)”.

Analyses were performed using SPSS 21.0 IBM ® and GraphPad Prism 5.0 GraphPad ® and statistical significance was considered with *p* ≤ 0,05.

## Results

A total of 1131 patients with COVID-19 admitted to the ICU were included. Patients mean age was 52 ± 15,8 y/o., with a prevalence of males 60% (*n* = 678). Most common comorbidities included hypertension 63.8% (*n* = 722) and diabetes 23.7% (*n* = 269). A total of 49% (*n* = 556) of patients were taking angiotensin converting enzyme inhibitors (ACEi) or angiotensin receptor blockers (ARBs) prior to ICU admission. 16% (*n* = 185) of patients were on vasopressors and 19% (*n* = 220) were on mechanical ventilation at ICU admission (Table 1).

**Table 1:** Demographic data

Age (y/o)	10—19	2	,2%
	20—29	48	4,2%
	30—39	165	14,6%
	40—49	224	19,8%
	50—59	257	22,7%
	60—69	223	19,7%
	70—79	124	11,0%
	80—89	69	6,1%
	90—99	19	1,7%
Gender	Female	453	40,1%
	Male	678	59,9%
Hypertension	No	409	36,2%
	Yes	722	63,8%
Diabetes	No	862	76,2%
	Yes	269	23,8%
ACEi	No	1032	91,2%
	Yes	99	8,8%
ARB	No	705	62,3%
	Yes	426	37,7%
ACEi or ARB	No	575	50,8%
	Yes	556	49,2%
Cancer	No	1091	96,5%
	Yes	40	3,5%
COPD	No	1072	94,8%
	Yes	59	5,2%
CKD	No	1098	97,1%
	Yes	33	2,9%
Non- Invasive Ventilation	No	920	81,3%
	Yes	211	18,7%
Mechanical Ventilation	No	911	80,5%
	Yes	220	19,5%
Mortality	No	1004	88,8%
	Yes	127	11,2%
AKI	No	755	66,8%
	Yes	376	33,2%

The risk of AKI was 33% (*n* = 376), 78% (*n* = 293) of which did not require dialysis and 22% (*n* = 83) presented dialytic AKI. Overall mortality was 11% (*n* = 127), while for AKI patients, mortality rate was 21% (*n* = 80).

Significant risk factors for AKI development at univariate analysis were the following: comorbidities variables – age, diabetes, hypertension, ACEi or ARB use, chronic obstructive pulmonary disease (COPD) and chronic kidney disease (CKD); ICU admission variables: need for mechanical ventilation or vasopressors, serum potassium > 4.2 mEq/L, serum sodium < 140 mEq/L, pH < 7.35, pCo2 > 48 mmHg, c-reactive protein (CRP) > 8.7 mg/dL and lymphocytes < 720 (Table 2).

**Table 2:** Univariate analysis of risk factors for AKI development

		**Non AKI**		**AKI**		**X2 Pearson**	**Odds Ratio**	**IC 95%**	**IC 95%**	**Total**	
		**n**	**%**	**n**	**%**	**Sig**	**OR**	**Inferior**	**Superior**	**n**	**%**
**Age**	** < = 43**	225	29,8%	62	16,5%	0,000	2,2	1,6	2,9	287	25,4%
	** > = 43**	530	70,2%	314	83,5%					844	74,6%
**Gender**	**Female**	300	39,7%	153	40,7%	0,757	1,0	0,7	1,2	453	40,1%
	**Male**	455	60,3%	223	59,3%					678	59,9%
**Hypertension**	**No**	273	36,2%	136	36,2%	0,997	1,0	0,8	1,3	409	36,2%
	**Yes**	482	63,8%	240	63,8%					722	63,8%
**Diabetes**	**No**	596	78,9%	266	70,7%	0,002	1,6	1,2	2,1	862	76,2%
	**Yes**	159	21,1%	110	29,3%					269	23,8%
**ACEi**	**No**	681	90,2%	351	93,4%	0,077	0,7	0,4	1,1	1032	91,2%
	**Yes**	74	9,8%	25	6,6%					99	8,8%
**ARB**	**No**	439	58,1%	266	70,7%	0,000	0,6	0,4	0,7	705	62,3%
	**Yes**	316	41,9%	110	29,3%					426	37,7%
**ACEi or ARB**	**No**	351	46,5%	224	59,6%	0,000	0,6	0,5	0,8	575	50,8%
	**Yes**	404	53,5%	152	40,4%					556	49,2%
**Cancer**	**No**	732	97,0%	359	95,5%	0,206	1,5	0,8	2,9	1091	96,5%
	**Yes**	23	3,0%	17	4,5%					40	3,5%
**COPD**	**No**	725	96,0%	347	92,3%	0,008	2,0	1,2	3,4	1072	94,8%
	**Yes**	30	4,0%	29	7,7%					59	5,2%
**CKD**	**No**	739	97,9%	359	95,5%	0,024	2,2	1,1	4,4	1098	97,1%
	**Yes**	16	2,1%	17	4,5%					33	2,9%
**Non Invasive Ventilation**	**No**	648	85,8%	272	72,3%	0,000	2,3	1,7	3,1	920	81,3%
	**Yes**	107	14,2%	104	27,7%					211	18,7%
**Mechanical Ventilation**	**No**	679	89,9%	232	61,7%	0,000	5,5	4,0	7,6	911	80,5%
	**Yes**	76	10,1%	144	38,3%					220	19,5%
**Vasopressors**	**No**	691	91,5%	255	67,8%	0,000	5,1	3,7	7,2	946	83,6%
	**Yes**	64	8,5%	121	32,2%					185	16,4%
**Sodium**	** > 140**	472	73,8%	158	42,1%	0,000	3,9	2,9	5,1	630	62,1%
	** < = 140**	168	26,3%	217	57,9%					385	37,9%
**Potassium**	** < = 4,2**	311	48,7%	60	16,0%	0,000	5,0	3,6	6,8	371	36,6%
	** > 4,2**	328	51,3%	315	84,0%					643	63,4%
**pH**	** > 7.35**	393	62,2%	118	32,7%	0,000	3,4	2,6	4,4	511	51,5%
	** < = 7.35**	239	37,8%	243	67,3%					482	48,5%
**PCO2**	** < = 48.0**	440	70,0%	149	41,4%	0,000	3,3	2,5	4,3	589	59,6%
	** > 48.0**	189	30,0%	211	58,6%					400	40,4%
**Platelets**	** > 149**	512	77,6%	225	61,3%	0,000	2,2	1,7	2,9	737	71,8%
	** = < 149**	148	22,4%	142	38,7%					290	28,2%

Variables selected for the logistic regression model and inclusion in the final prognostic score were the following: age, diabetes, ACEis, ARBs, chronic kidney disease and hypertension (Table 3). After complete clinical evaluation, each variable is added to one another, divided by 17 and multiplied by 100, indicating the risk of developing AKI during ICU stay (COV-AKI Score) (Table 4).

**Table 3:** Multivariate analysis of risk factors for AKI development

	**Sig**	**Hazard Ratio**	**IC 95% Inf**	**IC 95% Inf**
Age > 43 y/o	< 0,0001	2,01	1,50	2,70
Diabetes	0,006	1,39	1,10	1,75
ACEi	< 0,0001	0,40	0,26	0,61
ARB	< 0,0001	0,41	0,31	0,53
CKD	0,024	1,76	1,08	2,88
Hypertension	0,034	1,34	1,02	1,76

**Table 4:** COV-AKI score

+	Age > 43 y/o	x	3,5
+	**Diabetes**	x	2,5
-	**ACEi**	x	4,0
-	**ARB**	x	4,0
+	**CKD**	x	3,0
+	**Hypertension**	x	2,5

**Fig. 1:**
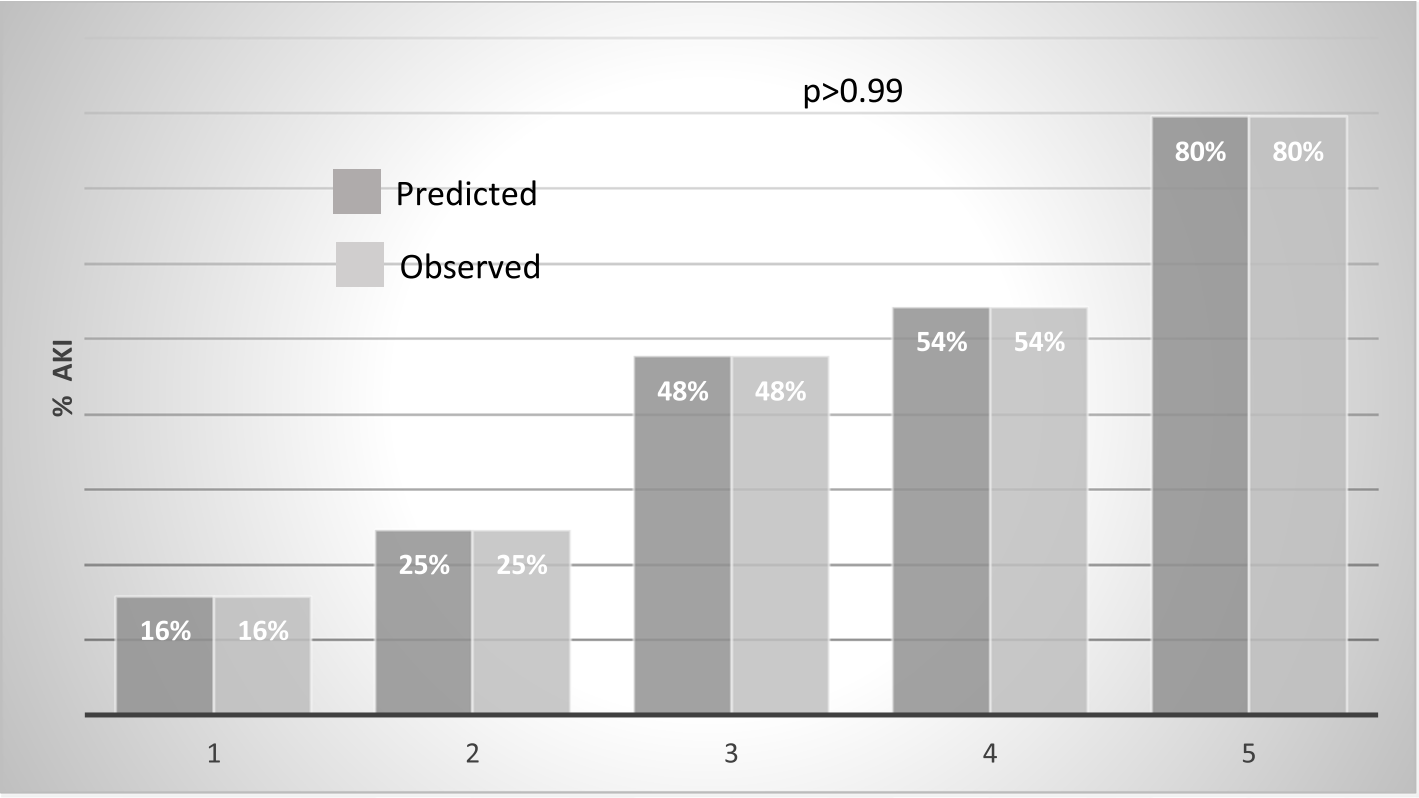
COV-AKI Score Calibration

**Fig. 2:**
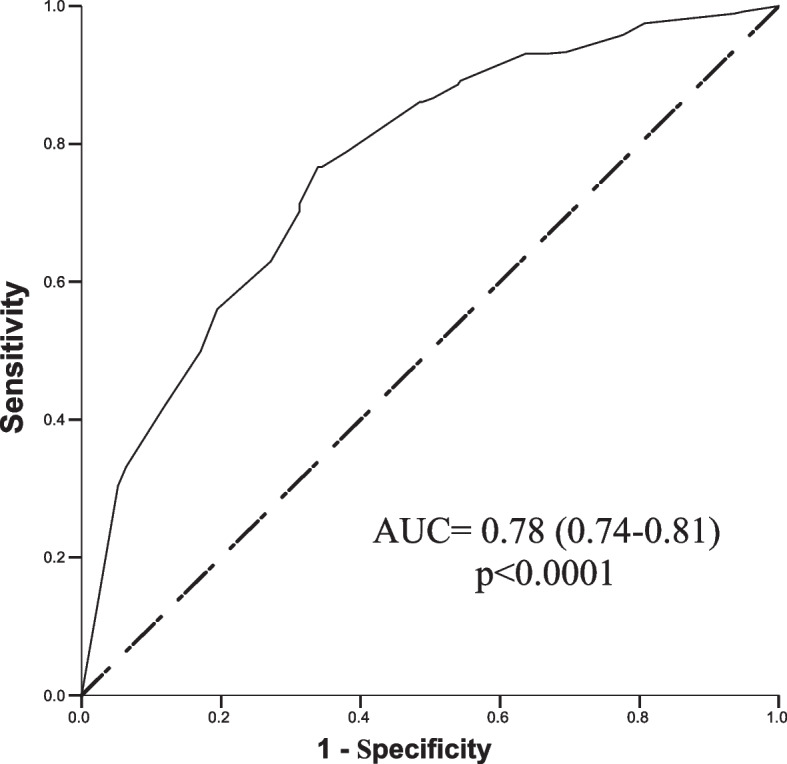
Discrimination of COV-AKI Score for AKI development

COV-AKI Score presented good performance in the calibration analysis using Hosmer–Lemeshow´s goodness-of-fit test with non-significant difference between the predicted and observed risk for development of AKI in COVID-19 patients during ICU stay (Fig. 1) Discrimination of COV-AKI Score was evaluated by analysis of the area under the receiving operating characteristic (ROC) curve, with an area under the curve (AUC) of 0.78 (0.74–0.81) (Fig. 2).

$$\mathrm{AKI risk }= [ (\mathrm{Age}>= 43 * 3.5) + (\mathrm{Diabetes }* 2.5) - (\mathrm{ACEI use }* 4.0) - (\mathrm{ARB use }* 4.0) + (\mathrm{Chronic kidney disease }* 3.0) + (\mathrm{Hypertension }* 2.5)] / 17*100$$

## Discussion

We developed a clinical score to predicit AKI development in COVID-19 patients admitted to the ICU. The variables included in the final model were age, diabetes, chronic kidney disease, hypertension and ARB or ACEi use. This clinical score will enable clinicians to predict the risk of AKI development in COVID-19 patients in order to minimize the risks of renal function decline and further clinical deterioration.

Recently, many clinical scores were developed to predict hospital mortality and ICU transfer or need for mechanical ventilation in COVID-19 patients, but none evaluated the risk for AKI development in these patients [28–33]. Wang et al. [34] described a clinical score to predict AKI development in a population of 389 COVID-19 patients, with an AKI incidence of 7.2%, much lower than observed in our study, where 33% of patients developed AKI, probably because we focused on ICU patients with more complex clinical presentations. Our AKI score has a very important feature when compared with other scores developed for AKI prediction in COVID-19 patients. We studied patients admitted to the Intensive Care Unit for the treatment of complications associated with COVID-19. In this way, our score is applied at the time of patient admission to the ICU to predict AKI development during ICU stay, thus selecting a more complex population of patients with worse prognosis and greater chance of developing organ dysfunctions, including kidney failure. Also, other authors did not determine a specific point in time where the score should be applied and combined different populations of patients in the same study, such as patients admitted to the emergency room, ward and the intensive care unit [17, 35–37]. Furthermore, Lu et al. [36], describes that the overall prediction performance by Area under Receiver Operating Charactheristic Curve (AUC) was good at day 0, and moderate at day -1 and -2, a finding with doubtful clinical significance, since at that moment (day 0) there is no sufficient time to adopt preventive measures that minimize the risk of developing AKI during hospitalisation.

The presence of advanced age and/or comorbidities, specifically diabetes and hypertension, were frequently included as important variables in many specific scores developed to predict complications in COVID-19 patients [29, 30, 38]. Our study also observed advanced age and diabetes or hypertension as variables associated with AKI development in COVID-19 patients and were included in the final model, probably reflecting a reduced renal reserve associated with the combination of inflammation and microvascular alterations affecting renal fuction and contributing to a greater incidence of AKI in these patients.

Chronic Kidney Disease (CKD) is a well known risk factor for AKI development in different clinical scenarios [39]. In our study, CKD was associated with AKI development in COVID-19 patients and also included in the final model, combining the negative effects of previously damaged kidneys with hyperinflammation, microthrombosis and direct infection of kidney cells seen in these patients.

At the beginning of COVID-19 pandemics, it was unclear if the use of ACEi or ARBs were associated with increased risk of complications and/or severe disease. Indeed, recent studies confirmed that when compared to untreated subjects, those using either ACEi or ARBs showed a similar risk of critical or lethal clinical course associated with COVID-19 infection [19, 21, 40–42]. ACE2 (Angiotensin-Converting Enzyme 2) is a extracellular transmembrane enzyme responsible for breaking down angiotensin II into angiotensin heptapeptide and also works as the main receptor for uptake of severe acute respiratory syndrome coronavirus 2 (SARS-COV-2) into the cell. ACEIs and ARBs act on the renin–angiotensin–aldosterone system (RAAS) reducing angiotensin II formation and consequently downregulating ACE2 expression and probably reducing binding of SARS-COV-2 into the kidney cells and protecting against the development of AKI in COVID-19 patients.

The comparison between our score and previous prognostic scores developed to predict complications such as need for mechanical ventilation, ICU transfer or death is not feasible because of different end points. In a recent review, Lombardi e cols [43]. studied the accuracy of 32 scores designed to predict ICU transfer or death and found an area under the receiver operating characteristic curve (AUC ROC curve) > 0.75 in only seven studies. Our score (COV-AKI Score) presented a good discriminative performance to predict AKI development in COVID-19 patients with an AUC ROC curve of 0.78. The COV-AKI Score can be easily calculated at the bedside, without the need for complex laboratory tests or clinical variables hard to compute on a daily basis, making it also appropriate for application in countries with limited resources.

The limitations of the present study are related to the lack of external validity to evaluate model performance in different settings and also limited to ICU patients, precluding any conclusion about the risk profile to AKI development in distinct scenarios such as wards or emergency departments.

## Conclusions

Our study identified the main risk factors for the development of AKI in patients with COVID-19 admitted to the Intensive Care Unit and also developed a prognostic score capable of identifying patients at high risk for AKI, facilitating the adoption of preventive measures that minimize the risk of this complication in COVID-19 patients.

## Data Availability

All data generated or analysed during this study are included in this article. Further enquiries can be directed to the corresponding author.
